# Dapagliflozin: A Role for Heart Failure Treatment in Dialysis?

**DOI:** 10.1016/j.ekir.2026.106494

**Published:** 2026-03-21

**Authors:** Bróna M. Moloney, Finnian R. Mc Causland

**Affiliations:** 1 Renal Division, Brigham and Women’s Hospital, Boston, Massachusetts, USA; 2 Renal Division, Harvard Medical School, Boston, Massachusetts, USA

See Clinical Research on Article 106355

Cardiovascular (CV) disease is the leading cause of death among patients with kidney failure requiring hemodialysis (HD), occurring in approximately 40% to 50% of this population. Around 40% of prevalent patients have a diagnosis of heart failure (HF), whereas approximately 25% of patients develop *de novo* HF after HD initiation (the latter have a 3-year survival of only 17%).^1^ Hospitalizations for volume overload are frequent (∼24%), and around half of 30-day readmissions are thought to be related to pulmonary edema.^2^ Such stark data highlight the burden of CV disease and HF among these patients and emphasize the need for testing new interventions to improve outcomes.

Sodium-glucose cotransporter 2 inhibitors (SGLT2i) heralded a paradigm-shift in the treatment of patients with CKD who were not on dialysis. Unparalleled data from multiple trials demonstrated the benefit of these drugs to reduce CKD progression, reduce CV morbidity and mortality, and improve quality of life.^3^^,^^4^ However, patients receiving HD were excluded from these trials, resulting in continued equipoise regarding their use in this population.

Some observational studies have suggested beneficial associations of SGLT2i use among patients receiving HD.^5^ Although it is tempting to extrapolate data from patients with CKD to those receiving HD, we should be cognizant of failed past experiences (e.g., recently with mineralocorticoid receptor antagonists).^6^ Further, as infection is the second leading cause of death among patients receiving HD, valid concerns related to the risk of urogenital infections with SGLT2i among patients with HD remain. From a mechanistic perspective, some have raised concerns that decreased or absent diuretic activity of SGLT2i among patients with oligo-anuria may limit their CV effects. However, this narrow mechanistic view overlooks potential kidney-independent pathways, such as attenuation of oxidative stress, anti-inflammatory activity, and improved cardiac function.^5^

In this respect, the dapagliflozin cardiovascular effects on end-stage kidney disease-2 trial (DARE-ESKD-2) by Barreto *et al.*,^7^ among patients receiving dialysis (HD and peritoneal), is an important contribution. DARE-ESKD-2 was a prospective, randomized, open-label study, conducted across 3 dialysis centers that randomized 80 (79 included in final analysis) individuals to dapagliflozin or standard care, with blinded end point assessment. The primary outcome was the change in N-terminal pro-B-type natriuretic peptide (NT-proBNP) concentration from baseline to 24 weeks. Secondary outcomes included the change in Kansas City Cardiomyopathy Questionnaire (KCCQ) score and the 6-minute walk test distance. The primary outcome result was reported as a treatment effect of −155 pg/ml (95% confidence interval: −327 to −33 pg/ml) for the baseline-adjusted median change in NT-proBNP, but the *P*-value from the rank-based analysis of covariance was 0.065. Higher (improved) KCCQ scores were also noted for dapagliflozin-treated individuals versus standard of care, but did not reach statistical significance; there was no meaningful effect on the 6-minute walk test distance. To better interpret these results, it is imperative to conduct a deeper exploration of the trial design and participants.

The population was relatively young (mean age 56 years), had a median dialysis vintage of 3.5 years (85% received HD), and over 50% had significant residual urine output (median 600 ml/day). Only 18% had a history of diabetes mellitus. Despite 68% having a history of HF, the median NT-proBNP was only 626 pg/ml. Overall, this suggests a relatively ‘healthy’ group, at least compared with a typical US population receiving HD. As such, the included patients may have been at relatively low risk of HF, making it harder to observe any beneficial signals from a pharmacologic intervention.

The intervention was dapagliflozin at a dose of 10 mg daily, the same dose used in the dapagliflozin and prevention of adverse outcomes in chronic kidney disease trial (DAPA-CKD; the latter allowed patients to remain on treatment after dialysis initiation).^8^ However, the present comparator was standard of care, as opposed to a placebo. As outlined by the authors, this open-label design is 1 of the major limitations of the trial, as the lack of a placebo predisposes to a substantial risk of bias. For example, the dialysis prescription management was left to the patient’s own nephrologist, a pragmatic choice that emulates real-world practice and minimizes study burden, but one that may have increased the risk of bias and Hawthorne effect. Although the end point measurements were performed in a blinded fashion, this does not mitigate the issues of lack of patient and investigator blinding.

In this Phase 2 design, the study appropriately considered surrogate outcomes, including NT-proBNP, KCCQ, 6-minute walk test distance, and echocardiographic parameters of volume overload. Although exercising extreme caution with the small sample size and aforementioned caveats, there were some interesting signals observed, despite the overall null results for the primary and secondary outcomes. For example, the change in NT-proBNP narrowly missed statistical significance and was in the direction of benefit, consistent with other trials of SGLT2i in nondialysis populations. Similarly, a 7-point increase in KCCQ scores was observed; though nonsignificant, the 95% confidence intervals mostly included positive changes (i.e., improvement).

Of course, there are challenges with biomarkers of volume among patients receiving HD. In particular, the timing of measurement becomes critically important. The longer interdialytic period is well recognized as the “high-risk” period for patients requiring HD,^9^ thus, assessing volume-sensitive biomarkers during the shorter interdialytic period, as occurred in this study, may limit the ability to detect more extreme changes. Similarly, consistency in the timing of measurement in relation to HD (i.e., pre- or post-HD) is also important. In this respect, it also becomes challenging to pool data from patients receiving peritoneal dialysis with that of patients receiving HD, as the former generally have less variability in their volume status over the course of a typical dialytic week. Although the collection of HF symptoms is welcome in patients receiving dialysis, KCCQ has not been validated specifically in such patients. Similarly, concerns relate to the use of 6-minute walk test among patients receiving HD, who are a relatively frail population, where comorbid conditions other than HF or hypervolemia may play an important role in lower exercise tolerance.

So where do we go from here? On the one hand, one could be unenthusiastic about this trial based on the null primary result. On the other hand (despite the limitations), one could exercise cautious enthusiasm and pursue SGLT2 inhibition in a larger study with a higher-risk population. However, much work remains to be done to promote and optimize trials in this area. In particular, more research effort is needed in relation to volume-related adverse events – how they should be defined, ascertained, adjudicated; are there acceptable surrogates, and which quality of life instruments are appropriate and valid in this population? Should we consider standardized protocols for dialysis prescriptions, including dialysate sodium and ultrafiltration rates, which may help to reduce treatment heterogeneity? Enrichment strategies for volume-related adverse events could also be considered to recruit participants at a higher risk of events (enhancing the ability to detect treatment effects). Given the high baseline rates of CV events and death observed among patients receiving dialysis, the performance of ‘hard’ outcomes trials seems feasible, but patient-centered trial designs need to evolve to maximize the ability to recruit and retain these patients who are often already ‘fatigued’ from frequent healthcare encounters.

In summary, the question of whether SGLT2is are beneficial for patients with kidney failure remains unanswered. The DARE-EKSD-2 study highlights the urgent need to generate such evidence via event-driven trials focused on clinically meaningful outcomes in these patients. Thankfully, larger-scale trials are already underway (e.g., NCT05374291) and will hopefully provide more robust evidence to improve the lives of this high-risk and understudied population.

## Disclosures

BMM reports funding via the Ben J. Lipps Research Fellowship Grant from the American Society Nephrology. FRMcC reports research fees from Novartis, Lexicon, AstraZeneca, and NIH, paid directly to his institution; consulting fees from Aquapass, GSK, and Zydus Therapeutics; expert witness fees from Rubin-Anders Scientific, Covington & Burling LLC; travel/speakers fees from Bayer and Global Learning Collaborative; he serves as a DSMB member for the TwoPlus trial; his spouse reports research funding from Lexicon Pharmaceuticals paid directly to her institution; consulting fees from Vera Therapeutics and Alexion; serves on DSMB for AstraZeneca.
